# New evidence for the Ontong Java Nui hypothesis

**DOI:** 10.1038/s41598-023-33724-9

**Published:** 2023-05-25

**Authors:** M. L. G. Tejada, T. Sano, T. Hanyu, A. A. P. Koppers, M. Nakanishi, T. Miyazaki, A. Ishikawa, K. Tani, S. Shimizu, K. Shimizu, B. Vaglarov, Q. Chang

**Affiliations:** 1 Research Institute for Marine Geodynamics, Japan Agency for Marine-Earth Science and Technology, Yokosuka, 237-0061 Japan; 2 Department of Geology and Paleontology, National Museum of Nature and Science, Tsukuba, 305-005 Japan; 3 College of Earth, Ocean and Atmospheric Sciences, Oregon State University, Corvallis, OR 97331 USA; 4 Graduate School of Science, Chiba University, Chiba, 263-8522 Japan; 5 Department of Earth and Planetary Sciences, Tokyo Institute of Technology, Tokyo, 152-8550 Japan; 6 Graduate School of Science and Engineering, Chiba University, Chiba, 263-8522 Japan; 7 Kochi Institute for Core Sample Research, Japan Agency for Marine-Earth Science and Technology, Kochi, 783-8502 Japan

**Keywords:** Ocean sciences, Planetary science, Solid Earth sciences

## Abstract

The formation of the Ontong Java Nui super oceanic plateau (OJN), which is based on the model that the submarine Ontong Java Plateau (OJP), Manihiki Plateau (MP), and Hikurangi Plateau (HP) were once its contiguous fragments, could have been the largest globally consequential volcanic event in Earth’s history. This OJN hypothesis has been debated given the paucity of evidence, for example, the differences in crustal thickness, the compositional gap between MP and OJP basalts and the apparent older age of both plateaus relative to HP remain unresolved. Here we investigate the geochemical and ^40^Ar-^39^Ar ages of dredged rocks recovered from the OJP’s eastern margin. Volcanic rocks having compositions that match the low-Ti MP basalts are reported for the first time on the OJP and new ~ 96–116 Ma and 67–68 Ma ^40^Ar-^39^Ar age data bridge the temporal gap between OJP and HP. These results provide new evidence for the Ontong Java Nui hypothesis and a framework for an integrated tectonomagmatic evolution of the OJP, MP, and HP. The isotopic data imply four mantle components in the source of OJN that are also expressed in present-day Pacific hotspots sources, indicating origin from (and longevity of) the Pacific Large Low Shear-wave Velocity Province.

## Introduction

**Figure 1:**
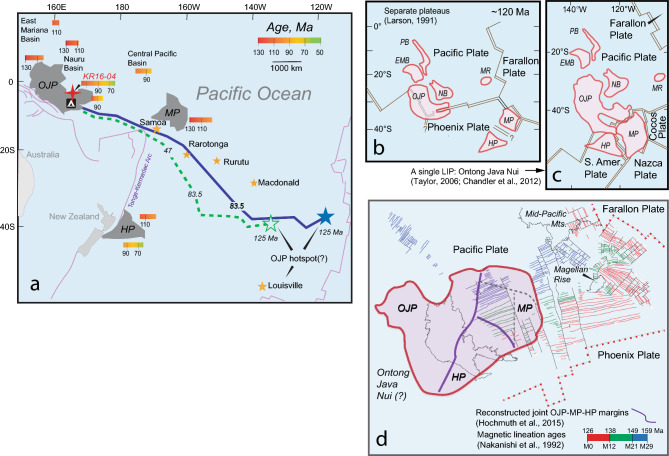
Present-day and reconstructed position of the Ontong Java Nui rifted fragments. (**a**) Line drawing of the present location of Ontong Java (OJP), Manihiki (MP), and Hikurangi (HP) plateaus comprising the postulated rifted fragments of the Ontong Java Nui (OJN) super plateau. The red cross shows the study area, and the orange stars mark present day hotspot locations. Two reconstructed OJP hotspot paleopositions and transport paths to present-day location are shown by green dashed and blue solid flowlines^4^. Reconstructed paleoposition of (**b**) separately emplaced ^2,7^ and (**c**) combined^3,4,7^ OJP, MP, and HP forming a single superplateau are illustrated. (**d**) The inferred OJN configuration (pink area) relative to OJP, following Hochmuth et al.^5^, superimposed on reconstructed OJP position (light solid outline) and magnetic lineations at 125 Ma^1^. Maps and magnetic lineations were drawn using Generic Mapping Tool version 6^8^. Bold purple solid lines are the conjoined rifted margins of the OJP, MP and HP. Dashed gray lines are inferred fragmentation boundaries within MP^5^. Also indicated by color scale in panel a) are age ranges for OJP, MP, and HP, as well as the surrounding basins^9–18^.

**Figure 2:**
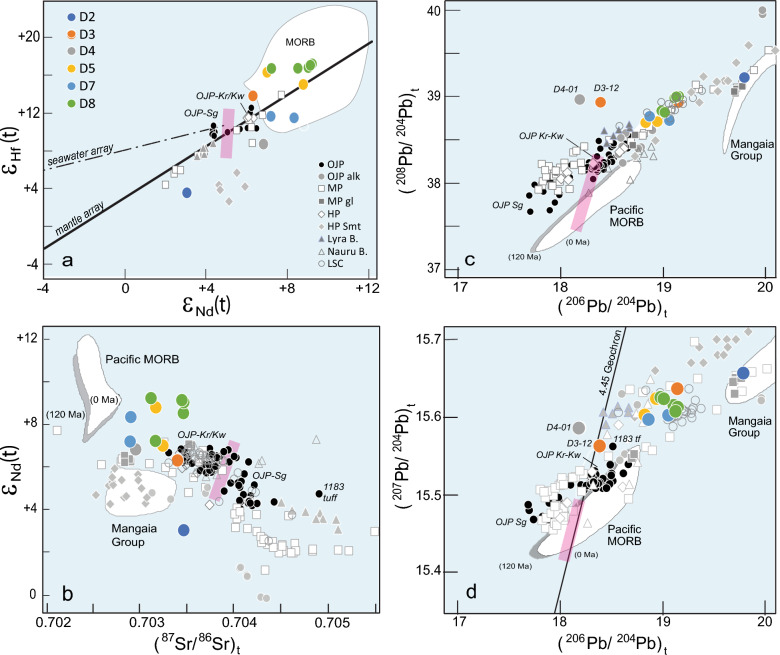
Binary isotope data plots for KR16-04 samples. (**a**) ε_Nd_(t) vs. ε_Hf_(t); (**b**) (^87^Sr/^86^Sr)_t_ vs. ε_Nd_(t); (**c**) (^206^Pb/^204^Pb)_t_ vs. (^208^Pb/^204^Pb)_t_; (**d**) (^206^Pb/^204^Pb)_t_ vs. (^207^Pb/^204^Pb)_t_. Two samples, D3-12 and D4-01, have anomalous (^206^Pb/^204^Pb)_t_ values, owing to alteration of parent isotope U and possibly La and Nd due to phosphatization, which also caused unreasonably high ε_Nd_(t) values of + 24 for D3-12 (not shown; Supplementary Materials). Added for comparison are data for Ontong Java Plateau and associated younger alkalic rocks^9–11,19,20,23^, Manihiki Plateau^13–15,21,22^, Hikurangi Plateau^14^, Nauru Basin^16^, Lyra Basin^24^, and Louisville Seamount Chain (LSC)^25^. Other data fields shown for reference include Pacific ridge basalts (MORBs) ^26–29^ and Mangaia Group^30,31^. Geochemical boundary (pink band) between Hawaii’s Loa (OJP-Sg side) and Kea (OJP-Kr/Kw side) trends is drawn for reference ^32,33^. Estimated 120 Ma positions are shown for MORB (shaded) and Mangaia Group in Pb-Nd–Sr plots assuming the only changes in the source have been radioactive decay and ingrowth and assuming the following ^87^Rb/^86^Sr, ^147^Sm/^144^Nd, and ^238^U/^204^Pb values^11^: 0.02, 0.24, and 5 for the MORB source and 0.054, 0.20, and 22 for the Mangaia Group source. To remove interlaboratory bias, all Nd and Sr isotope data are normalized to accepted values of ^87^Sr/^86^Sr = 0.710251 for NBS987^34^ and ^143^Nd/^144^Nd = 0.511858 for La Jolla Nd or 0.512115 for J-Ndi standard^35^ before plotting (Table S4). Analytical errors on data in this study are smaller than the size of the symbols. Abbreviations: Sg = Singgalo-type basalt; Kr/Kw = Kroenke and Kwaimbaita-type basalt; gl = glass; alk = alkalic; R. = ridge; B. = basin; Smt. = seamount.

The Pacific Ocean contains the greatest number and largest volcanic edifices known as oceanic plateaus (Fig. 1a). Despite their size, oceanic plateaus are little studied due to their remote locations and formation during the Cretaceous Magnetic Quiet Period (CMQP)^1^ when the Earth’s magnetic pole stayed the same for the longest span of geologic time. Formation of these plateaus during CMQP means no magnetic anomalies constrain their original location. Thus, available paleoreconstructions evolved from one showing Ontong Java (OJP) and Manihiki Plateau (MP) forming separately^2^, to another where OJP, MP, and Hikurangi Plateau (HP) are loosely to tightly connected to form a single super plateau^3–5^ (Fig. 1b-d). The latter configuration was first proposed by Taylor^3^ and later coined the Ontong Java Nui (OJN) by Chandler et al.^4^, which postulates that OJP, MP, and HP are rifted fragments of one super plateau (Fig. 1c). If the OJN hypothesis is proven, then it represents the largest (59–90 million cubic km) magmatic event recorded in Earth’s history^6,7^. Such an event would have had significant consequences for the paleoenvironment. However, whether such a jigsaw-like reconfiguration occurred is still debated since there are parts of the puzzle that do not match^7^. Proponents of the hypothesis struggle to explain the contrasting crustal thicknesses among them. In addition, ^40^Ar-^39^Ar age range for the HP (96–118 Ma) indicates that this plateau was emplaced later than the main plateau building phase of both OJP and MP, for which previously dated samples indicate formation prior to 120 Ma (Fig. 1a)^9–15^. Available geochemical data from MP basement show a bimodal range of correlated ^206^Pb/^204^Pb and TiO_2_ values from unradiogenic (17.788–18.091) for high-Ti basalts (TiO_2_ > 0.9 wt%) to radiogenic (18.691–20.035) for low-Ti basalts (TiO_2_ < 0.9 wt%) that contrast markedly with the limited spread in OJP and HP data (Fig. 2)^9–11,13–15,19–22^. The absence of plateau basalts with radiogenic MP-like Pb isotopic composition in OJP (17.697–18.675)^9–11,19,20^ and HP (17.93–18.62)^14^ is one of the important missing pieces of evidence for the OJN hypothesis. In addition, the high MgO and depletion in moderately incompatible trace elements characteristic of the low-Ti MP basalts are unlike any found so far on OJP and HP^13,21^.

**Figure 3:**
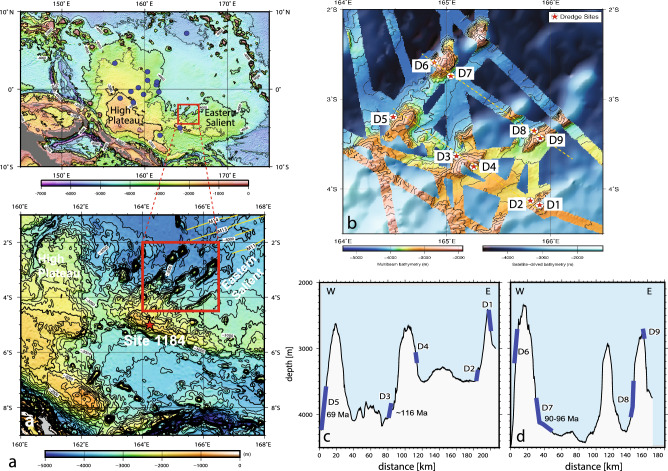
Maps and bathymetric profiles of the study area. (**a**) Map of the OJP and inset showing the KR16-04 survey area along the Eastern Salient just north of Ocean Drilling Program Site 1184 (red star) generated using GMT version 6^8^ and MB-System version 5.7.8^36^. Yellow lines are magnetic lineations M11 to M13^1^. The background bathymetric data are from SRTM15 + : GLOBAL BATHYMETRY AND TOPOGRAPHY AT 15 ARCSECONDS https://topex.ucsd.edu/WWW_html/srtm15_plus.html. (**b**) Detailed bathymetry and KR16-04 dredge locations; (**c**) and (**d**) Bathymetry profiles along the cross-section lines in (**b**), with ^40^Ar-^39^Ar age data (Fig. S2). The multibeam data, except for those from KR16-04, were obtained from JAMSTEC and NCEI/NOAA databases. JAMSTEC: Data and Sample Research System for Whole Cruise Information (DARWIN); NCEI/NOAA: NCEI Bathymetric Data (https://www.ncei.noaa.gov/maps/bathymetry/).

In this paper, we report the geochemical and ^40^Ar-^39^Ar dating results from volcanic rocks dredged from the unexplored eastern margin of the OJP (Fig. 3). The study area lies along the Eastern Salient, which is the reconstructed center where OJP, MP, and HP were conjoined (Fig. 1c-d)^3–5^. If the OJN hypothesis were correct, we could expect coeval flows with compositions comparable to those found on the MP at this location. Indeed, we recovered new basalt types that were previously not found on the OJP and their isotopic compositions match those of the MP basalts, suggesting similar mantle source. Dating results also indicate that some of the unusual OJP flows erupted contemporaneously with MP and HP. This discovery of matching source composition and age further binds the OJP, MP and HP fragments together and supports the OJN super plateau hypothesis.

## Results

### Bathymetry and^40^Ar-^39^Ar ages

During KR16-04 cruise, a geological survey and nine dredging operations were conducted in the eastern margin of the OJP^37^ (Figs. 1a, 3). The area covers the northern margin of the Eastern Salient of OJP, from which four subparallel NE-trending ridges protrude (Fig. 3a, b). These ridges form extended spurs of the Eastern Salient that run subparallel to magnetic lineations M11-M13 (136–138 Ma)^1^. Dredges D1, D2, and D4 retrieved samples from two apparent post-erosional features on top of an older volcanic platform (Fig. 3c). Dredge D3 was recovered from downslope of this older platform. Most of the seamounts are elongated along NE direction, subparallel to the magnetic lineations. One seamount (≤ 50 km by ≤ 20 km, ~ 2000 m high), on which dredges D8 and D9 were taken, is similar to the ridge-type seamounts found on top of HP^14^ (Fig. 3b, d). The two seamounts on which dredges D5, D6, and D7 were conducted are part of the same older and eroded northeast trending ridge (Figs. 3b-d).

Groundmass ^40^Ar-^39^Ar dating of samples from D3 and D7 yielded a plateau age of 115.56 ± 0.36 Ma for D3-02 and mini plateau ages of 96.10 ± 0.30 Ma (D7-10) and 90.46 ± 0.33 Ma (D7-20), respectively (Fig. 3 c-d; Supplementary Material). Considering that the plateau age from D3-02 is defined by 55% of ^39^Ar released, we consider this an age of moderate quality; the two mini-plateau ages from D7 samples with < 30% of the ^39^Ar released only provide low quality estimates for the eruption ages. This is because groundmass dating of submarine basalts are likely affected by undetectable alteration that increases the amount of ^39^Ar lost by recoil during sample irradiation, making ages appear older, the age spectra often more discordant, and if plateaus develop these are typically much shorter^38,39^. No plateaus developed for D8 samples and this dredge thus did not yield any useful age information. Nevertheless, the D3-02 age of 116 Ma overlaps well with the main plateau building episodes on OJP, HP, and MP^9–15^. This older age is consistent with the lower stratigraphic location of dredge D3 downslope on an old volcanic platform (Fig. 3c). This result indicates that the older platforms underbuilding the NE-trending ridges mapped during KR16-04 all must have formed during the main eruptive phase of OJP.

In contrast, plagioclase separates from two samples from dredge D5, on the same NE-trending ridge where dredge D7 is located, yielded younger plateau ages of 68.52 ± 0.31 Ma and 68.88 ± 0.28 Ma for sample D5-02 and 67.46 ± 0.33 Ma and 67.07 ± 0.34 Ma for sample D5-31. These dates are defined by narrow plateaus (< 60% of ^39^Ar released) that are not entirely flat, indicating some alteration disturbance and potentially older than the true age for these seamount samples^38,39^. However, overlapping results between repeat analysis and the small (1.42 Ma) age difference between D5-02 and D5-31 provide some reassurance to the quality of these ages, which at 68–69 Ma are markedly younger than the ~ 116 Ma D3-02 age.

### Bridging the compositional gap between OJP, HP, and MP

**Figure 4:**
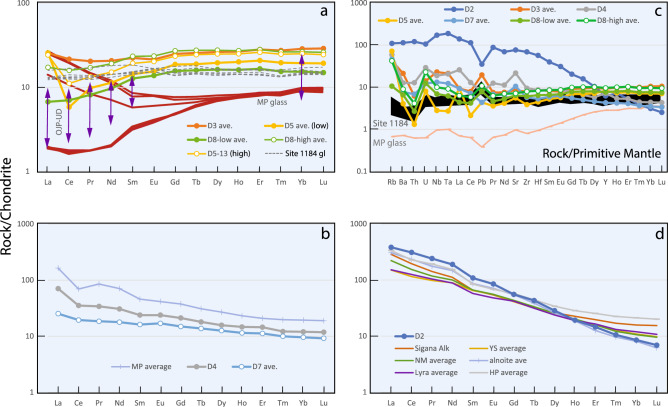
Trace element patterns for KR16-04 samples. Chondrite-normalized rare earth elements (**a**, **b**, **d**) and primitive mantle-normalized trace element (**c**) concentration plots for KR16-04 samples. Data for low-Ti Manihiki Plateau glasses (MP gl)^21^ and Ontong Java Plateau glasses from Site 1184^12^ and ultradepleted melt inclusions (OJP-UD)^40^ are shown for comparison. (**a**) Manihiki-like low- and high-Ti basalts from D3, D5, and D8; (**b**) transitional MORB-like basalts from D4 and D7; (**c**) incompatible trace element patterns showing three types of contrasting shapes consistent with rare earth element plots in (**a**), (**b**), and (**d**); (**d**) OIB-like basalt from D2 compared with OJP (Sigana, Youngers Series, North Malaita, Lyra Basin, alnöites) and HP (seamounts) alkalic rocks^10,14,23,41^. Abbreviations: YS = Younger Series; NM = North Malaita.

The volcanic rocks recovered from the study area range in composition from tholeiitic, transitional, to alkalic basalts, as illustrated well by their trace element patterns (Figs. 4, S4). Samples from D5 and D8 display a rare-earth element (REE) pattern characterized by a decreasing abundance of middle REE (MREE) to light- REE (LREE) but increasing amount of highly incompatible elements. This shape contrasts with the flat REE patterns of compositionally dominant Kwaimbaita-type OJP tholeiites, represented by volcanic glasses recovered by drilling at International Ocean Drilling Program (IODP) Site 1184, located ~ 200 km upslope to the south of the dredge area^9–12,42^. Samples from D4 and D7 have lower concentrations of heavy rare-earth elements (HREE) than D5 and D8 and increasing abundances from MREE to LREE to highly incompatible elements, resembling transitional mid-ocean ridge basalt (T-MORB) pattern. A sample from D2 shows the highest MREE to LREE and lowest HREE abundances relative to those of other samples, forming steeply sloped pattern characteristic of ocean island basalts.

Isotopic data for most (18 out of 21) of the dredge samples form tight clusters in Sr–Nd-Hf-Pb isotope binary plots, suggesting that they originated from the same source (Fig. 2; Table S3). Two samples, D3-12 and D4-01, have anomalous (^206^Pb/^204^Pb)_t_ values that plot away from the cluster. This may be attributed to recent seawater addition of parent isotope U, which leads to over age-correction of measured values (Figs. S3-S4; Table S2; Supplementary Material). Alteration of La and Nd abundances due to phosphatization of D3-12 resulted in unreasonably high ε_Nd_(t) value of + 24, which is much higher than values reported for depleted mantle or any oceanic basalts. The same alteration effect could also explain the slightly higher Nd isotope ratios for a given Sr and Hf isotope value of some samples relative to others from the same dredge (Fig. 2a–b). In contrast, the D2 sample possesses different radiogenic Pb isotopic compositions, much like those of the alkalic glasses from the central Danger Island Trough (DIT) on MP and from other seamounts on HP.

Interestingly, our new isotope data show the following features (Fig. 2, Table S3): 1) they form clusters that are different from those of the previously studied tholeiites of OJP and the younger alkalic rocks from Lyra Basin and the Solomon Islands found along the western and southern OJP margins; 2) they plot in the more radiogenic Pb isotope compositions, (^206^Pb/^204^Pb)_t_ = 18.834–19.157, than OJP data (17.697–18.675) and match those of the DIT and Suvorov Trough (or low-Ti MP) tholeiites (18.691–20.035); and 3) they overlap with the Louisville seamounts (LSC) data, especially in Pb-Pb isotope plots. This is the first time that rocks with such composition are recovered from the OJP, bridging the long-standing isotopic compositional gap between the plume head and its inferred Louisville plume tail, and between MP and OJP^9,20,25^.

## Discussion

The ~ 96–116 Ma ages obtained from our study encompass the main emplacement of the OJP, MP, and HP and the surrounding Nauru Basin (NB) and Central Pacific Basin (CPB), which were sampled by ocean drilling (Fig. 1a). IODP Site 1184 on the OJP’s Eastern Salient is located ~ 200 km upslope to the south of the dredge area and recovered volcaniclastic rocks that contain “fresh” glasses and basalt clasts (Fig. 3a). Although of poor quality due to severe submarine alteration, their ages of 74 ± 1.6 Ma and 123.5 ± 3.6 Ma were determined on plagioclase separates from these volcanic clasts^12^. Drilling at Deep-Sea Drilling Program (DSDP) Site 462 also sampled the igneous complex consisting of an upper series of younger ~ 100 to 75 Ma basalt sills and a lower series of older ~ 130 to 115 Ma basalt sheet flows and rare pillow lavas in the Nauru Basin (NB) just 1100 km north of the study area^16^. Beyond NB and farther to the northeast, 100–105 Ma pillow basalts were drilled at DSDP Site 169 and dolerite sills dated 90 ± 3.5 and 97 ± 2.5 Ma were found intruding in the overlying sediments in the Central Pacific Basin (CPB)^18^. On the MP, plateau phase volcanic rocks previously sampled by dredging and remotely operated vehicle have ^40^Ar/^39^Ar age range of 126.0 ± 1.5 to 122.9 ± 1.6 Ma, while ocean drilling recovered 117.0 ± 4.7 Ma to 117.3 ± 8.0 Ma basalts at DSDP Site 317^13–15^. Single crystal analyses of feldspars from basalts and a gabbroic sample recovered by dredging along the Rapuhia scarp on the HP also gave an age range of 96.3 ± 5 to 118.4 ± 4 Ma^14^. Thus, the ~ 96–116 Ma ages determined on samples from the KR16-04 dredge sites D3 and D7 fit well with the dates obtained on MP, HP, and adjacent NB and CPB (Fig. 1a). They are also consistent with new plagioclase-derived 106–116 Ma ages for samples from the other OJP drill sites on the High Plateau (Fig. 3a)^43^. These results suggest that the platforms where dredges D3 and D7 were taken formed during the main plateau building phase on OJP, MP, and HP. Notably, the 96–116 Ma ages overlap with the suggested ~ 86–118 Ma timing of the fragmentation of the OJN super plateau, which may have been accompanied by triple junction jumps and microplate formation, as well as seafloor spreading and sill intrusions, in the adjacent areas^3–5,16–18,44^.

The 67–69 Ma ages from D5, together with the uncertain age estimates obtained from D7 (~ 90 Ma), may indicate rejuvenated volcanism along pre-existing weaknesses after fragmentation, or pre-existing ridge axes in the surrounding basins (Figs. 1d, 3a)^1,5,44^. Samples from seamounts on different parts of the HP also gave 87–99 Ma and a younger age of 66.9 ± 6.0 Ma. The HP seamounts are considered shield and post-erosional stages based on morphological features in some of them, with the elongation of ridge-type seamounts attributed to volcanism along extensional faults^14^. For the KR16-04 seamounts, we infer that crustal weaknesses along pre-existing ridge axes and transform faults coupled with OJP’s passage over the South Pacific “hotspot highway”^45^, may have resulted in volcanic flare ups along plateau margins from 90–44 Ma^10,41^.

The discovery of tholeiitic rocks with LREE-depleted signatures and isotopic compositions like the MP low-Ti basalts is a significant result of this study. In all drilling sites on the main plateau and the Solomon Islands, the dominant Kwaimbaita-type OJP tholeiites display flat patterns, except for all but the most incompatible elements, as shown by Site 1184 glasses (Fig. 4a, c)^9–12,42^. Previously, the LREE-depleted basalt types were only reported from the Danger Island and Suvorov troughs on MP^13,21^, although ultra-depleted melt inclusions in Kroenke-type basalts hinted that such composition also exists for OJP (Fig. 4a)^40^. The LREE-depleted patterns but higher concentrations of the D5 and D8 samples indicate that they were derived by fractional crystallization from such ultra-depleted melts. The fact that these LREE-depleted OJP tholeiites also have radiogenic Pb isotopic composition that match with those previously found only in MP provides one of the important missing pieces of evidence in the OJN hypothesis. A common origin from re-melting of a highly depleted plume mantle source containing small and variable amounts of a recycled HIMU (high time-integrated ^238^U/^204^Pb ratio)-like component could account for the geochemical features of MP^21^ and, by extension, for the geochemically similar OJP basalts. These results lend support for the OJN hypothesis or at least a common mantle source across all three plateaus.

The combined data for the OJP, HP, and MP to date indicate the presence of three isotopic groups for the main plateau stage and a fourth group for the younger alkalic rocks with HIMU-type composition (Fig. 2). This requires a four-component source for the OJN: Singgalo- and Kwaimbaita-type expressed in OJP and HP basalts, the LSC-type composition expressed in low-Ti MP basalts and the HIMU-type expressed in alkalic rocks. The LSC isotopic signature epitomizes the focal zone (FOZO) mantle, a common component in Pacific OIBs, and the inference is that it represents ancient, depleted mantle^47^. Its expression in MP and now OJP basalts, as well as its persistence in the LSC for at least the past ~ 70 million years, suggest it is a long-lived feature and may represent lower mantle material entrained in plumes^47,48^.

**Figure 5:**
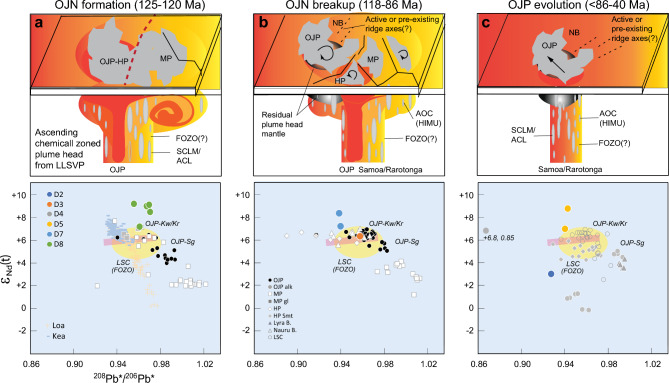
Cartoon depicting the inferred tectonomagmatic evolution of the OJN and the OJP. Reconstruction of OJN and surrounding NB and CPB^1–5,16,17^ is illustrated. Each column represents specific time interval and includes a map view (top), inferred mantle source beneath (middle), and ^208^Pb*/^206^Pb* vs. ε_Nd_(t) plots^33^ for emplaced basalt flow composition in each period (bottom). The top panel in a) reflects the Pacific Large Low Shear-wave Velocity Province (LLSVP) at the core-mantle boundary region where the plume generation zones (PGZ, thick red dashed line) intersect the reconstructed OJN paleoposition after Torsvik^50^. (**a**) Formation of OJN at 120–125 Ma upon impact on oceanic lithosphere of a surfacing chemically zoned plume head, with plume axis consisting of hot, primitive mantle material represented by OJP Kroenke/Kwaimbaita-type basalts^19,46^ and entrained lower mantle material with focal zone “FOZO” mantle signature represented by low-Ti MP basalts^15,22^. Ellipses represent associated recycled ancient continental crust (medium size) and subcontinental lithospheric mantle (largest size) expressed in Singgalo-type and high-Ti MP tholeiites, respectively^15,22,23,49^. The smallest ellipses represent more fusible recycled oceanic crust characterized by high time-integrated ^238^U/^204^Pb ratio (HIMU)^47^. (**b**) OJN breakup between I18 to ~ 86 Ma^3–5,44^, accompanied by emplacement of younger Kroenke-Kwaimbaita type basalts on OJP and HP above the hot plume axis. A thermochemical root, interpreted as residual plume head mantle beneath OJP^52^, formed after the initial plume impact and may have been separated from the main plume axis due to plate reorganization. (**c**) Between 86 and 40 Ma, MP and HP have dispersed away from OJP. Only OJP is shown depicting post-plateau volcanism along its western (Lyra Basin), southern (Younger Series, North Malaita Alkalics, Sigana Basalts), and eastern margins (KR16-04 dredge area) as it moved over the persistent plume mantle upwelling from LLSVP where several hotspots are located, such as Samoa, Rarotonga, and Rurutu^45,53,54^. Volcanism along pre-existing ridge axes or extension faults tapped underlying FOZO-type mantle with increasing contributions from more fusible recycled altered oceanic crust (HIMU) as the degree of melting decreased.

Previous works on OJP, HP, and MP favor origins from large degree melting above a surfacing plume head^9–11,14,15,19–22^. Within the context of the plume head model, the available data from the three oceanic plateaus suggest two isotopically resolvable mantle components within the plume head, as indicated by the OJP-HP (Kwaimbaita-Kroenke-type) and OJP-high-Ti MP (Singgalo-type) plateau basalts. The Kwaimbaita component is attributed to hotter ancient near-primitive mantle, owing to its near bulk-Earth Pb isotopic composition close to the Geochron^19,46^. The Singgalo component is ascribed to recycled ancient continental and subcontinental materials^15,22,23,49^. The Kwaimbaita-Kroenke and Singgalo components also simulate the Kea and Loa trends for Hawaii (Figs. 2, 5)^32,33^, and indicate their dual, potentially zoned existence since ~ 120 Ma in the plume head mantle source of the OJN.

The isotopic variation expressed in the OJN source may be rooted from the seismologically delineated Large Low Shear-wave Velocity Province (LLSVP) at the base of the mantle. It has been proposed that the LLSVP could be the ultimate source of hotspot volcanism in the Pacific, like Hawaii and the Polynesian volcanoes^32,33^. Paleoreconstruction also shows that OJN, when projected down to the core-mantle boundary region, straddles the edge of the LLSVP and shows the OJP at the inner side and the MP at the outer side (Fig. 5a)^50^. This scenario may explain the isotopic variations within the OJN source: an upwelling thermochemically zoned plume head dominated by Kwaimbaita component rises from the inner side of the LLSVP, entraining lower mantle (FOZO) at the margins, with both containing recycled (Singgalo and HIMU) materials. It is also consistent with suggestions that ambient lower mantle at the margins of the LLSVP is entrained in the upwelling plumes^47,48^.

Considering the range of ages, the compositional variation among volcanic rocks from the OJP, MP, HP and surrounding basins may also reflect the spatiotemporal contributions of the mantle source components in response to the changing tectonic configurations and degree of melting (Fig. 5). The bulk of the OJP may have been emplaced away from a ridge, based on evidence for pre-existing MORB lithosphere beneath^51,52^. In contrast, MP may have formed above a ridge, which led to its subsequent fragmentation along pre-existing spreading axes (Fig. 1d)^1,5^. Given the mantle source scenario above, contrasting tectonic setting may have led to OJP (and HP) tapping more of the plume axis material, and MP incorporating more of entrained lower mantle (Fig. 5a, b). The isotopic similarity of volcanic rocks from D3 and D8 with those of NB and low-Ti MP basalts suggests similar origin to the MP basalts (Fig. 5b).

A cartoon summarizing the tectonomagmatic evolution and possible plume mantle source structure during emplacement of the OJN is presented in Fig. 5. (1) OJN formed at 120–125 Ma upon impact on oceanic lithosphere of a surfacing chemically zoned plume head, with plume axis consisting of hot, primitive mantle material represented by OJP Kroenke/Kwaimbaita-type basalts^19,46^ and entrained lower mantle material with focal zone “FOZO” mantle signature represented by low-Ti MP basalts (Fig. 5a)^15,22^. It is postulated that the FOZO-type mantle dominated the fringes of the upwelling mantle and melted to a large degree along ridges and transform faults. (2) Breakup of the OJN occurred between 118 to ~ 86 Ma, accompanied by emplacement of younger Kroenke-Kwaimbaita type basalts on OJP and HP above the hot plume axis (Fig. 5b). The FOZO mantle component was tapped by volcanism and intrusions in surrounding basins above ridges and transform faults or transient triple junctions that later led to fragmentation of the OJN, forming KR16-04 basalts and possibly Nauru and Central Pacific basin basalts and sills at the same time. A thermochemical root, interpreted as residual plume head mantle beneath OJP^52^, formed after the initial plume impact and may have been separated from the main plume axis due to plate reorganization. (3) Between 86 and 40 Ma, MP and HP have dispersed away from OJP (Fig. 5c). The younger 68–69 Ma volcanism forming seamounts D5 and possibly D4 may have continued to tap the FOZO-type mantle as OJP crossed over the Pacific LLSVP that has been feeding the “hotspot highway" since 120 Ma^45,53^. LSC, the FOZO signature remains a component in Samoan lavas^54^. The fact that Samoan and Rarotongan isotopic overprints manifest in younger alkalic volcanism on the OJP is consistent with the plateau’s passage over these hotspots on its way to its present location (Fig. 1a)^10,24^. Increasing contribution from more fusible recycled oceanic crust (HIMU) as the degree of melting decreased may explain the composition of D2 and similar OJP, MP and HP alkalic basalts.

In conclusion, while not discounting the alternative hypothesis that OJP and MP were emplaced separately, the discovery of new composition and ages of volcanic rocks from the eastern margin of the OJP provides a breakthrough for the proposed OJN hypothesis. The geochemical data fills the compositional gap between the OJP-HP and MP that finally tie them with the Louisville mantle plume and the deep seismic structure underlying present-day Pacific hotspots. Although the overall sequence of the OJP emplacement relative to its two counterparts still needs to be refined, the results enable an integrated view of OJN’s tectonomagmatic evolution from its emplacement and its subsequent fragmentation, including the occurrence of post plateau volcanism on the OJP. Given that there are still uncertainties about the paleolocation of each plateau relative to each other, more geophysical surveys and geochemical and geochronological data are required from this remote region in the Pacific to further test the Ontong Java Nui hypothesis.

## Methods

### ^40^Ar-^39^Ar dating

Dredging recovered mostly tholeiitic and a few alkalic basalts along the northeast trending ridges in the eastern margin of the OJP (Fig. 3). Twelve least altered plagioclase-phyric samples from D2, D3, D5, D7, and D8 were selected for ^40^Ar-^39^Ar dating (Supplementary Table S1). However, only two samples from D5 yielded plagioclase separates while the rest had either few or tiny plagioclase phenocrysts. For the latter samples, groundmass was used for dating. ^40^Ar-^39^Ar plateau ages were obtained by incremental heating technique at the Oregon State University Argon Geochronology Laboratory using ARGUS VI-D mass spectrometer. Measured ages were reduced using ArArCALC^38^ and normalized using Fish Canyon Tuff standard age of 28.201 ± 0.046 Ma^55^ and the age equations and decay constants from Min et al.^56^.

### Major and trace elements analysis

Based on the thin section descriptions, 68 less altered samples from seven dredge hauls (D2, D3, D4, D5, D7, D8 and D9) were selected and then major and selected trace elements were determined using a Rigaku ZSX Primus II X-ray fluorescence (XRF) spectrometer at the National Museum of Nature and Science (NMNS), following the procedures of Sano et al.^57^. The selected samples were cut into ~ 1 cm wide slices and washed with running hot water (~ 50 °C) for seven days to desalinate the samples by removing any traces of seawater. The desalinated slices were crushed into 1 ~ 3 mm diameter chips, and only fresh-looking (dark grey to grey color) least altered chips of each sample were handpicked. The least altered grains were then washed ultrasonically, twice in alcohol (10 min) and twice in distilled water (10 min), dried for > 12 h in an oven at 110 °C and then ground into powder in an alumina mill. Loss on ignition (LOI) values were determined prior to major and trace element analyses by weighing ~ 0.5 g of powder on a Mettler Toledo dual balance system before and after heating the powder at 1025 °C for 4 h in an electric muffle furnace. Fused glass beads for major element analysis were prepared after LOI determination using a lithium tetraborate flux (10:1 dilution of sample). For trace element analysis, ~ 4.0 g of powder was pressed into a pellet by a 15-ton force from a hydraulic press.

After XRF analysis, 25 samples with less altered (LOI < 6 wt %), except for 1 sample (D5-07), were selected for measurement of a larger range of trace elements by inductively coupled plasma-source mass spectrometry (ICP-MS). Trace element compositions were determined using a quadrupole Agilent 7700 × ICP-MS instrument at NMNS and the procedures described by Sano et al.^58^. Prior to ICP-MS analysis, the samples were digested using a HF-HClO_4_-HNO_3_ acid attack with final dissolution in 2% HNO_3_ plus 0.1% HF solution and spiked with ^115^In and ^209^Bi. These elements were added to standardize the signal for the ICP-MS measurements. Internal precision and external reproducibility are typically better than 1% and 3%, respectively.

### Pb-Nd–Sr-Hf isotope analysis

Isotopic data for Pb, Nd, Sr, and Hf were acquired using the analytical facilities at Japan Agency for Marine-Earth Science and Technology (JAMSTEC) following detailed analytical procedures described in Miyazaki et al.^59^.

All sample powders were leached prior to Sr, Nd, Hf, and Pb isotope analyses. Approximately 1 g of each powder sample was weighed into acid-washed 30 mL Savillex® Poly tetra-Fluoroethylene-co-perfluoro Alkyl vinyl ether (PFA) beakers. Then, samples were mixed with 20 mL of 6 M HCl and kept at 100 °C for 2 h. After the supernatant was decanted, 20 mL of 6 M HCl and 1 mL of concentrated HF were added and kept at 100 °C for 30 min.

For Sr and Nd isotope analysis, ~ 120 mg of leached powder was decomposed with a 1:3 mixture of 12 M HClO_4_ and 20 M HF followed by digestion in a 1:3 mixture of 12 M HClO_4_ and 6 M HCl, and then in 6 M HCl, respectively. For Pb isotope analysis, ~ 150 mg of leached powders was decomposed with a 1:4 mixture of 15 M HNO_3_ and 20 M HF followed by digestion in 8 M HBr.

The Sr–Nd–Hf separation was carried out using an AG50W-X8 cation ion exchange resin (Bio Rad, California, USA), along with Sr-spec and Ln-spec resins (Eichrom Tec. Inc., Illinois, USA). The Pb separation was performed using 0.1 mL of AG1-X8 anion exchange resin (Bio Rad, California, USA). Column separation procedures were conducted with the fully automated open-column chemical separation system COLUMNSPIDER, developed by JAMSTEC and HOYUTEC Co., Ltd. (Kawagoe, Japan)^60^. The total procedural blanks for Sr, Nd, Hf, and Pb were less than 5, 3, 13, and 10 pg, respectively.

Sr and Nd isotope ratios were measured with a thermal ionization mass spectrometer (TIMS) using a Triton TI (Thermo-Finnigan, Bremen, Germany). The Sr and Nd isotope ratios measured were normalized to ^86^Sr/^88^Sr = 0.1194 and ^146^Nd/^144^Nd = 0.7219, respectively, to correct for mass fractionation. The mean ^87^Sr/^86^Sr value in the standard reference material (SRM) from the National Institute of Standards and Technology (NIST) (SRM 987) was 0.710242 ± 0.000027 (errors in two standard deviations (2SD), n = 6) and the mean ^143^Nd/^144^Nd value in the JNdi-1 standard was 0.512092 ± 0.000021 (2SD, n = 5) during the analyses, equivalent to ^143^Nd/^144^Nd = 0.511835 for the La Jolla Nd standard using the factor of 1.000503^35^. Hf isotope ratios were measured at JAMSTEC using a multiple collector-inductively coupled plasma mass spectrometer (MC-ICPMS) (Neptune; Thermo Scientific, Bremen, Germany) at JAMSTEC. ^173^Yb and ^175^Lu peaks were monitored during the measurement to correct for interference from ^176^Yb and ^176^Lu on the ^176^Hf peak and were always undetectable. The instrumental mass fractionation was determined by the ^179^Hf/^177^Hf ratio and normalized to 0.7325. Repeated measurements of the JMC-475 Hf standard solution during the analyses resulted in a mean ^176^Hf/^177^Hf ratio of 0.282152 ± 0.000011 (2SD, n = 22). Pb isotope ratios were determined using the same MC-ICPMS. The mass fractionation of the Pb isotope was corrected using Tl as an external standard and a standard bracketing method using NIST SRM 981 as a standard. Repeated measurements of ^206^Pb/^204^Pb, ^207^Pb/^204^Pb, and ^208^Pb/^204^Pb using NIST SRM 981 yielded values of 16.9274 ± 0.0013, 15.4818 ± 0.0013, and 36.6664 ± 0.0055 (2SD, n = 19), respectively. We reported ^206^Pb/^204^Pb, ^207^Pb/^204^Pb, and ^208^Pb/^204^Pb ratios normalized to reference NIST SRM 981 values of 16.9416, 15.5000, and 36.7262, respectively^61^.

For determinations of parent-daughter isotopes ratios, the concentrations of Rb, Sr, Sm, Nd, Lu, Hf, Pb, Th, and U in the aliquot solution for Sr and Nd isotope analysis were determined by solution nebulization quadrupole inductively coupled plasma-mass spectrometry (ICP-MS) ((iCAP Qc ThermoFisher Scientific, Bremen, Germany) at JAMSTEC. The analytical repeatability and accuracy were estimated to be better than 2% and 5–7%, respectively^62^.

## Supplementary Information

Supplementary Information 1.

Supplementary Information 2.

## Data Availability

All data generated or analysed during this study are included in this published article [and its supplementary information files].
