# Supplementary material for: New evidence for the Ontong Java Nui hypothesis
Source: Sci Rep. 2023 May 25;13:8486. doi: 10.1038/s41598-023-33724-9 (PMC10213030; doi:10.1038/s41598-023-33724-9)
Supplement: Supplementary file 2 — Supplementary Information 2. [file 41598_2023_33724_MOESM2_ESM.docx]

**SUPPLEMENTARY INFORMATION TO**

**New evidence for the Ontong Java Nui hypothesis**

MLG TEJADA^1*^, T SANO^2^, T HANYU^1^, A KOPPERS^3^, M NAKANISHI^4^, A ISHIKAWA^5^, T MIYAZAKI^1^, K TANI^2^, S SHIMIZU^6^, K SHIMIZU^7^, B VAGLAROV^1^and Q CHANG^1^

^1^Institute for Marine Geodynamic, Japan Agency for Marine-Earth Science and Technology, Yokosuka 237-0061, Japan

^2^Department of Geology and Paleontology, National Museum of Nature and Science, Tsukuba 305-005, Japan

^3^College of Earth, Ocean and Atmospheric Sciences, Oregon State University, Corvallis, OR 97331, U.S.A.

^4^Graduate School of Science, Chiba University, Chiba 263-8522, Japan

^5^Department of Earth and Planetary Sciences, Tokyo Institute of Technology, Tokyo 152-8550, Japan

^6^Graduate School of Science and Engineering, Chiba University, Chiba 263-8522, Japan

^7^Kochi Institute for Core Sample Research, Japan Agency for Marine-Earth Science and Technology, Kochi 783-8502, Japan

**Contents:**

1. **Supplementary Text** file describing the samples, alteration types and geochemical signatures attributed to alteration effects

**2. Supplementary Figures**

Figure S1: Representative photos of samples from each dredge site.

Figure S2: ^40^Ar-^39^Ar age spectra for dated samples.

Figure S3: Major elements vs. loss-on-ignition (LOI) and alteration test plots for KR16-04 samples.

Figure S4: Primitive mantle-normalized trace element patterns for KR16-04 samples.

**3. Supplementary Tables**

Table S1: Summary of ^40^Ar-^39^Ar dating results.

Table S2: X-ray fluorescence major and trace elements data and inductively coupled plasma mass spectrometry (ICP-MS) trace element abundances for KR16-04 dredge samples. Additional columns indicate geochemical affinities of KR16-04 basalts based on TiO_2_ contents and trace element patterns.

Table S3: Sr-Nd-Hf-Pb isotopes data and parent-daughter trace element abundances for KR16-04 samples.

Table S4: Compiled Pb-Nd-Sr-Hf isotopes and parent-daughter elements data for Ontong Java Plateau.

**Supplementary Text:**

**Samples**

Dredging during KR16-04 cruise recovered 68 volcanic rocks consisting of aphyric and sparsely phyric to porphyritic basalts (Fig. S1). Samples from dredges D2 and D7 are sparsely olivine+plagioclase-phyric, while those from D3 are plagioclase-phyric. The samples with higher amounts of plagioclase±pyroxene phenocrysts were recovered from D5, with up to 22 modal % plagioclase, while those from D7 and D8 are mostly aphyric to sparsely olivine- or plagioclase-phyric. All experienced variable degrees of submarine alteration, as indicated by greenish gray to rusty, as well as bleached, light brown spots and discoloration bands of samples. These variable degrees of alteration are reflected in the ^39^Ar-^40^Ar age spectra of dated samples (Fig. S2).

**Geochemical signatures attributed to alteration effects**

Loss on ignition (LOI) values of 1.87 to 12.23 indicate low to high degrees of alteration and water interaction (Fig. S3). Some show marked enrichment in P_2_O_5_ and CaO, indicating phosphatization (D2-03, D5-07, and D8-40) while others experienced K_2_O enrichment and/or loss of CaO (D3-12, D3-13), leading to elevated K_2_O/CaO ratios. These samples, together with those having more than 6 wt% LOI that show marked decreases in SiO_2_, CaO, Na_2_O, and MgO and elevated K_2_O and MnO are considered too altered to give meaningful magmatic compositions and are not considered further for petrogenetic interpretations. Most of these samples belong to D5 and D9 (D9-02, D9-05). However, the TiO_2_ content, which is believed to be more alteration resistant, suggests that three compositional types were recovered (Table S2; Fig. S3): low-Ti basalts from D5 and D8 (TiO_2_ ~1 wt%) medium-Ti basalts from D3, D4, D5, D7 and D8 (1.4 to 2.0 wt% TiO_2_) and high-Ti basalts from D2, D5 and D9 (>2.0 wt% TiO_2_). However, the high TiO_2_ contents of D5 and D9 samples with >6 wt% LOI do not correlate with high total alkali contents as expected for alkalic rocks. Most of these samples display low (<1 wt%) Na_2_O, which could be an effect of alteration. These altered, high-Ti samples from D2, D5, and D9 also have high concentrations of immobile incompatible elements suggesting alkalic affinities but display irregular patterns (Fig. S4). All samples share the slightly positive Ta-Nb anomaly with Kwaimbaita-type Site 1184 and Hikurangi basement basalts. However, there is an apparent negative Ta-Nb anomaly in the D4 and D5 patterns relative to elevated La and U concentrations (Fig. S4). In addition to the U spikes, they have anomalous (La/Nb)n values of >1, which could be an effect of alteration on the light rare earth elements during incipient phosphatization. Similar coupled elevation of La and U is observed in altered basalts that experienced phosphatization from Nuugurigia atoll on the OJP High Plateau (Hanyu et al., 2017) and Ojin Rise seamounts (Tejada et al., 2016).

Sample D13-12 yielded anomalously high ^143^Nd/^144^Nd ratio of 0.513868 (ε_Nd_ = +24.1), which is much higher than values reported for depleted mantle or any oceanic basalts. This sample also has the highest K_2_O and lowest CaO, indicating that the anomalously high Nd isotope ratio can be attributed to alteration effect and not a magmatic signature. This sample, together with D4-01, also has elevated U concentration and ^238^U/^204^Pb ratio even after leaching, indicating U addition by seawater alteration. Consequently, these two samples have the most radiogenic ^206^Pb/^204^Pb ratios (Table S3). Therefore, age-correction to initial values results in lower ^206^Pb/^204^Pb ratios for their given ^208^Pb/^204^Pb ratios relative to other samples (Fig. 2). These results suggest that U uptake took place more recently than the eruption age such that calculated initial magmatic values are over-corrected.

**Supplementary References:**

Hanyu, T., Tejada, M.L.G***.,*** Shimizu, K., Ishizuka, O., Fujii, T., Kimura, J-I., Chang, Q., Senda, R., Miyazaki, T., Hirahara, Y., Vaglarov, B.S., Goto, K.T., Ishikawa, A., 2017. Collision-induced post-plateau volcanism: Evidence from a seamount on Ontong Java Plateau: Lithos 294-295, 87-96.

Tejada, M. L. G., Geldmacher, J., Hauff, F., Heaton, D., Koppers, A. A. P., Garbe-Schönberg, D., Hoernle, K., Heydolph, K., and Sager, W. W., 2016. Geochemistry and age of Shatsky, Hess, and Ojin Rise seamounts: Implications for a connection between the Shatsky and Hess Rises. *Geochimica et Cosmochimica Acta*, **185**, 2, 302-327. <http://dx.doi.org/10.1016/j.gca.2016.04.006>

Supplementary Figures and Captions

Figure S1: Representative photos of samples from each KR16-04 dredge site.

Figure S2: ^40^Ar-^39^Ar age spectra for dated KR16-04 samples.

Figure S3: Major elements vs. loss-on-ignition (LOI) and alteration test plots for KR16-04 samples.

Figure S4: Primitive mantle-normalized trace element patterns for KR16-04 samples.
